# Functional and Aesthetic Periorbital, Ocular Adnexal and Ocular Surface Changes Linked to GLP-1 Receptor Agonists

**DOI:** 10.3390/jcm14248792

**Published:** 2025-12-12

**Authors:** Dimitrios Kapantais, Panagiotis Tsoutsanis

**Affiliations:** 1 Department of Ophthalmology, Northern Care Alliance NHS Foundation Trust, Rochdale OL12 0NB, UK; panagiotis.tsoutsanis@nca.nhs.uk; 2 Faculty of Medical and Human Sciences, The University of Manchester, Manchester M13 9PT, UK

**Keywords:** glucagon-like peptide-1 receptor agonists, periocular fat loss, aesthetic changes, ocular surface, orbit, adnexal

## Abstract

**Background/Objectives:** Glucagon-like peptide-1 (GLP-1) and glucose-dependent insulinotropic polypeptide (GIP) receptor agonists have revolutionised obesity and type 2 diabetes management through effective weight loss and metabolic regulation. However, their increasing use has led to reports of adverse aesthetic and functional effects, particularly affecting facial and ocular tissues. **Methods:** A comprehensive literature review was conducted in October 2025 across PubMed, Embase, and Medline using the terms “GLP-1 receptor agonist,” “Ozempic face,” “facial lipoatrophy,” “ocular surface disease,” “orbital fat,” and related combinations. Studies reporting facial, periorbital, orbital, or ocular surface changes associated with GLP-1 or GLP-1/GIP receptor agonists were included. Reference lists were screened to identify additional sources. **Results:** Evidence suggests that GLP-1 and dual GLP-1/GIP receptor agonists may contribute to rapid facial volume loss, dermal fat atrophy, and periocular hollowing—collectively termed “Ozempic face.” The mechanism is multifactorial, involving both weight-loss-related fat depletion and potential modulation of adipocyte differentiation. Ocular surface improvements have been observed in some studies. Radiologic data demonstrate preferential superficial midface fat loss, informing potential aesthetic correction strategies. **Conclusions:** GLP-1-based therapies, while clinically effective, can result in perceptible adnexal and periocular changes with aesthetic and functional implications. Awareness of these effects is crucial for multidisciplinary management. Future prospective studies are warranted to clarify mechanisms and guide individualised reconstructive and rejuvenative interventions.

## 1. Introduction

Glucagon-like peptide 1 (GLP-1) and glucose-dependent insulinotropic polypeptide (GIP) are gastrointestinal hormones that mediate the incretin effect after eating, which enhances glucose-stimulated insulin secretion when nutrients move through the gastrointestinal tract. GLP-1 and GIP exert their action by activating the GLP-1 (GLP-1R) and GIP (GIPR) receptors, respectively, which are expressed in the pancreatic endocrine islets, the gastrointestinal tract, the cardiovascular system, the brain, the kidneys, and immune cells [1].

While they both promote insulin secretion from pancreatic β-cells, GLP-1 and GIP exert distinct effects on glucagon secretion from pancreatic α-cells. GIP stimulates glucagon release during hypoglycaemia, whereas GLP-1 suppresses glucagon secretion during hyperglycaemia. Furthermore, GIP directly enhances lipogenesis, while GLP-1 indirectly promotes lipolysis. GLP-1 receptors in the gastrointestinal system reduce gastric emptying, while the GLP-1 receptors in the nervous system stimulate satiety [2].

All these actions help maintain healthy fat cells, limit abnormal fat accumulation, and boost adiponectin production and secretion from adipocytes. Overall, the coordinated activity of these two incretin hormones supports metabolic balance by preventing both hyperglycaemia and hypoglycaemia, improving lipid profiles, and lowering cardiovascular risk in individuals with type 2 diabetes and obesity [3,4].

The frequency of obesity in adults has doubled, while the rates in adolescents and children have quadrupled since 1990. The prevalence of adult obesity increased in 188 countries, with the highest increase in the female population of Sub-Saharan Africa and in the male population of Central Europe, Brunei, the USA, and Polynesia, while the highest rise in both sexes was noted in the Middle East, Caribbean, and North Africa [5]. According to the World Obesity Federation’s 2025 Atlas, the global number of adults affected by obesity is expected to rise by over 115% between 2010 and 2030, increasing from 524 million to 1.13 billion [6]. The WHO’s definition of obesity is a BMI equal to or greater than 30, while overweight is defined as an individual with a BMI equal to or greater than 25 [5]. Obesity and a higher-than-optimal BMI have been shown to increase risk for diabetes, several types of cancer, and death caused by noncommunicable diseases worldwide [7,8,9]. As a result, in recent years, more medications have been developed, aiming to reduce obesity and all the adverse effects associated with it.

Medications targeting GLP-1R exist, such as liraglutide (3.0 mg) and semaglutide (2.4 mg). Available in oral and injectable formulation, and, more recently, GLP-1R-GIPR co-agonists, such as tirzepatide (at 10 mg or 15 mg), are all recommended on the American Diabetes Association (ADA) Professional Practice Committee guidance for obesity and weight management for the prevention of type 2 diabetes [10]. Several studies have shown higher efficacy of GLP-1R-GIPR co-agonists compared to individual agonists, in weight loss and management of type 2 diabetes, while offering a similar safety profile, although the need for personalised treatment selection according to patient characteristics has been highlighted [11,12,13].

Over the years, this exponential increase in GLP-1R and GLP-1R-GIPR agonist use has led to various patients experiencing a variety of side effects in different organ systems. While there are several review studies reporting the effects of GLP1-RA and/or GIP agonists on generalised facial aesthetic changes and review studies that assess the ocular effects of these medications, to the best of our knowledge, there are no review studies that focus on the ocular adnexal and ocular surface effects that these medications can cause. In this study, we have performed a comprehensive literature review to investigate the effects of such medications on the periocular areas, orbit, and ocular surface and their management from the perspective of an oculoplastics specialist.

## 2. Materials and Methods

A comprehensive literature review was performed in October 2025 on PubMed, Embase, and Medline.

A broad search strategy was developed, combining Medical Subject Headings (MeSH) and free-text terms. The primary keywords and their combinations included the following, among others: “GLP-1 receptor agonist”, “GLP-1RA”, “Ozempic face”, “facial lipoatrophy”, “ocular surface disease”, “orbital disease”, “periorbital fat”, “dry eye”, and “orbital fat”. Boolean operators (“AND”, “OR”) were used to refine the search and capture studies across various terminologies and related anatomical or pharmacological contexts.

Articles were included if they discussed facial or periorbital morphological changes, mechanisms of soft-tissue atrophy, implications for oculoplastic or aesthetic surgery, or management strategies following GLP-1-associated weight loss. Both clinical and experimental studies, case series, systematic reviews, and relevant narrative reviews were considered.

The reference lists of all included studies were manually screened to identify additional relevant articles not identified in the initial database search. Only English-language publications available in full text were included. Duplicates were removed, and data from each article were extracted and synthesised qualitatively to identify recurring themes, surgical strategies, and emerging recommendations.

A comprehensive narrative review approach was selected rather than a formal systematic review because the topic—facial and periorbital changes associated with GLP-1RA use—is emerging and heterogeneous, with limited high-level evidence and significant variation in study design, terminology, and reported outcomes. The available literature consists mainly of case reports, small series, and conceptual papers, making meta-analysis or structured synthesis impractical. This broader, integrative method allowed the inclusion of relevant clinical, anatomical, and surgical insights to provide a contextual and practice-oriented overview suitable for guiding future research and clinical management.

## 3. Results

### 3.1. Effects of GLP1 Withdrawal or Cessation

Studies have shown that many patients who reach their target weight with GLP-1 receptor agonists eventually stop treatment, creating an urgent need to understand what happens afterwards. Clinical trials indicate that weight tends to return quickly once therapy is discontinued, with much of the regained weight being fat mass. Ongoing treatment with subcutaneous injections of semaglutide might be necessary [14]. However, these trial findings have limited real-world relevance since participants were blinded, and treatment withdrawal did not reflect typical clinical practice. More real-world research is needed to truly understand the effect of discontinuation of such medication [15].

### 3.2. Global Patterns in GLP1 Usage

The recent recommendations for GLP-1R agonists and GLP-1R-GIPR co-agonists for use in obesity have caused a global increase in their prescription and, thus, spending. In the U.S alone, total spending on GLP-1 receptor agonists rose by over 500% between 2018 and 2023, increasing from $13.7 billion to $71.7 billion. By 2023, semaglutide-based products (Ozempic, Rybelsus, and Wegovy) and tirzepatide (Mounjaro) together accounted for 70% of total spending [16].

Similarly, in the UK, there has been an upward trend in prescriptions of GLP-1 agonists such as semaglutide, especially since the ADA guidelines in 2022, and a recent increase in GLP-1R-GIPR co-agonists being prescribed since January 2024 [17].

### 3.3. GLP-1R Agonists and Other Pathologies

Diabetes control and obesity are not the only diseases that this class of medication has been tested on. Several studies have investigated the benefits of GLP-1R agonists and GLP-1R-GIPR co-agonists, both used for combating obesity, which is an increased risk factor or directly affects the course of various pathologies. These include cardiovascular diseases, obstructive sleep apnoea, polycystic ovarian syndrome, respiratory disorders, metabolic dysfunction-associated steatotic liver disease, and neurodegenerative diseases such as Parkinson’s, Alzheimer’s disease, or glaucoma [18,19,20,21,22,23,24,25].

### 3.4. GLP-1R Agonists GLP-1R-GIPR Co-Agonists and Anaesthetic Concerns

Since one of the main modes of action of GLP-1R agonist medications is to slow down gastric emptying, this introduces several risks to the administration of general anaesthesia, such as gastric content retention and pulmonary aspiration. Evidence has indicated that patients taking GLP-1R agonists may have a full stomach during surgery. Some authors recommend precautions, including a thorough review of medication use, enhanced monitoring, and preventive strategies, such as pre-anaesthesia gastric ultrasound and rapid sequence induction, while others note there is insufficient evidence to put forward definitive guidance regarding the ideal cessation period of such medication before elective surgery in order to lower risks [26,27]. The American Society of Anesthesiologists has published guidance that mentions that a patient should hold GLP-1 RA on the day of surgery if they take the medication daily and withhold it for one week if they take it weekly. They also suggested postponing an elective procedure if a patient has gastrointestinal symptoms like nausea or vomiting [28].

### 3.5. GLP-1R Agonists GLP-1R-GIPR Co-Agonists and Ophthalmic Involvement

Over the past few years, the main argument around ophthalmic involvement and side effects with the use of GLP-1R and GLP-1R-GIPR agonists has been regarding diabetic retinopathy (DR). Early clinical evidence from the SUSTAIN-6 trial, which investigated the cardiovascular outcomes in patients with type 2 diabetes taking semaglutide, showed a higher number of DR complications in patients taking it versus placebo. This result was attributed to the rapid blood sugar reduction, transiently exacerbating DR. More studies since then have produced mixed results, and the overall consensus now is that GLP-1R agonists do not significantly increase risk of DR development or worsening, but risk may vary according to individual patient characteristics [29,30,31,32].

Similarly to DR, there has been a lot of debate in the available literature regarding whether the use of GLP1-R agonists increases the risk of non-arteritic anterior ischemic optic neuropathy (NAION). Studies have shown mixed results, with earlier studies showing a positive association, whereas later studies showed no significant risk. Once again, patient characteristics matter, as the suggested mechanism of developing NAION secondary to GLP-1R agonist use is due to sudden improvements in blood sugar and weight, leading to an alteration in glucose metabolism and supply, potentially harming the metabolically demanding anterior portion of the optic nerve. Furthermore, these abrupt metabolic changes may temporarily disrupt the autoregulation of optic nerve perfusion, resulting in heightened vulnerability in people with small or crowded optic discs or low nighttime blood pressure to develop NAION [29,32,33,34,35].

There are also conflicting studies when the association of GLP-1R agonists and the development of age-related macular degeneration (AMD) is investigated. No concrete conclusions can be drawn at this point, as some studies report an increase in incidence, whereas others report protective effects [29].

Another aspect of ophthalmology where GLP-1R agonist involvement is investigated is that of glaucoma. These medications have shown neuroprotective effects to the retina by reducing oxidative stress and inflammation in preclinical studies. Moreover, certain observational studies have shown a reduction in the development of primary open-angle glaucoma (POAG) and ocular hypertension (OHT) in patients using GLP-1R agonists compared to other diabetic oral medications.

Overall, despite the promising results, the existing literature has several limitations, with all studies being retrospective and non-randomised. Future research with randomised case–control studies is needed to confirm the findings [22,29,36].

### 3.6. GLP-1R Agonists’ Effects on Periocular Area

In this review, we focused on the effects GLP-1R and GLP-1R-GIPR co-agonists have on the orbit, periorbital tissues, and the ocular surface.

Many individuals using GLP-1R agonists such as Ozempic, apart from their overall rapid weight loss, have had noticeable changes to their facial appearance. Most commonly, this involves a loss of volume, along with signs of accelerated ageing, sagging, and wrinkling of their skin. In fact, these effects have become so recognisable that the term “Ozempic face” has been coined by dermatologist Dr Paul Jarrod Frank in the United States to describe them. These effects can be frustrating for many individuals who receive treatment with GLP-1 RA to optimise their health and appearance [37]. There are some theories speculating that this loss of facial volume derives, in part, from the interaction GLP-1 peptides have with adipocyte differentiation mechanisms in the dermal white adipose tissue (dWAT). Dermal white adipose tissue (dWAT) is situated at the base of hair follicles and seeded throughout the dermis, and among other cells, it is associated with a strong contingent of preadipocytes—cells that look like fibroblasts and are capable of either returning to a fibroblast-like state or converting into different types of adipocyte-like cells.

While many plastic surgeons have patients asking for treatment post-GLP-1 RA treatment, there is a lack of scientific articles measuring the effect of GLP-1 on facial features [37].

There is an argument that the Ozempic face effect is caused by the rapid weight loss and not so much due to the usage of GLP-1 agonists, as it has not been shown that they can catabolize facial adipocytes. Individuals who lose a significant amount of weight will demonstrate a more pronounced facial wasting, which can include hollowness in the periocular area and loss of fat in the subcutaneous layer of the temples and cheeks [37,38,39,40]. Furthermore, there can be facial fat loss in the tear trough area [41]. Likewise, a potential loss of facial muscles can accentuate the premature aging effect [2,38].

The presence of GLP-1R agonists may be leading preadipocytes to revert to fibroblasts and myofibroblasts, leading to fibrosis and thinning of the extracellular matrix, and as a result, fat atrophy [42]. Furthermore, GLP-1R agonists have been shown to quickly inhibit the proliferation and fat-cell differentiation of adipose-derived stem cells (ADSCs) in vitro and lower their glucose uptake, leading to cell death [43].

A total of 65% of patients who were on GLP-1 treatment noticed facial volume loss, which was more pronounced in older individuals and in those who lost more than 10% of their body weight in less than six months [44].

The rapid weight loss causes the wrinkles to become more pronounced in tear troughs, temples, and cheeks. They can also change the size of the cheeks, affecting the balance of the facial proportions, and can cause loss of elastin, collagen, and essential nutrients in the skin [37].

In a retrospective study, Chen et al. assessed high-quality facial photographs of patients before and after their treatment with GLP1-RA in comparison to a control group of similar BMI, and performed several measurements of facial landmarks using an open-source image analysis software [45]. These measurements included the palpebral fissure height and width, the height of the upper eyelid, and the slant of the palpebral fissure. There was no statistically significant difference in the upper face distances of facial landmarks, but only a change in orolabial measurements of female participants. A limitation of the study was that all patients on GLP1 were recruited from an oculofacial clinic; therefore, they mainly had oculoplastic complaints, with half of them having upper dermatochalasis and half having ptosis [45].

Another concern of GLP1-RA is the concurrent excessive loss of muscle mass along with weight loss effects. Nutrient Stimulating Hormones (NuSHs) and Myostatin–activin pathway inhibitors (MAPi), which could be combined with GLP1-RA and GIP to preserve muscle mass and function, are under investigation, and their potential effects on facial musculature, which, in turn, can affect the facial contour and appearance, could be further clarified [38,46] (Table 1).

### 3.7. GLP-1R Agonists Effects on Orbit

While sunken eyes have been reported as part of the “Ozempic face”, there have been no studies assessing potential orbital changes, including any alterations of the orbital fat [47].

The orbital fat is a specialised type of deep facial white adipose tissue (WAT), which exists in the intraconal and extraconal orbital compartments and has multiple functions, including protecting vascular and neural structures and facilitating extraocular muscle movement [48,49]. The orbital adipose tissue has been shown to consist of small adipocytes separated with thick conjunctival septa anteriorly near the lacrimal gland and the extraocular muscles, while it consists of larger adipocyte cells separated by thin septa near the area of the optic nerve, and this configuration assists with their mechanical functions, offering support to the pulley system of the rectus muscles anteriorly and a freedom of movement for the optic nerve posteriorly [50]. It has been shown that the orbital fat has more of a resemblance to the buccal fat compared to the subcutaneous, as they both contain a higher concentration of collagen, mast cells, and endothelial cells [51].

Enophthalmos due to fat atrophy can be an age-related process and diagnosed as senile enophthalmos. However, it has been reported that even significant weight loss does not cause enophthalmos due to orbital fat reduction [52]. This was mainly based on the study of Mattacks et al., which was performed in 1985 on a guinea pig and showed that the orbital adipocytes can become larger to maintain the orbital volume after weight loss [53].

Regarding age-related enophthalmos, it has been proposed that descent of the Lockwood suspensory ligament and the inferior displacement of the globe can contribute to its development, which can, in turn, cause herniation of fat pads [54].

In a case report, a patient who lost a significant amount of 100 pounds of body weight after a diet over 3–4 years developed bilateral enophthalmos and was managed with orbital implant placement in the medial, lateral wall, and orbital floor. The enophthalmos was likely attributed to his massive weight loss and had deteriorated due to the large orbits of the patient [55].

In a prospective study, it was found that weight loss post-bariatric surgery caused a significant reduction in the intraocular pressure and a significant improvement in the retrobulbar and ocular blood flow as measured with Doppler ultrasound [56].

In a retrospective study that focused on orbital fat changes with age, measuring separate compartments of orbital fat on MRI scans, it was shown that individuals with higher BMI had increased orbital volume in all compartments, which caused a more anterior globe position [57].

However, Kuo et al. found a correlation between exophthalmos and obesity in Graves’ disease patients in Taiwan by measuring the globe position with a Hertel exophthalmometer, and the BMI and the orbital fat were removed during decompression surgery. The patients who underwent orbital decompression had significantly higher BMI than the general population, and a significantly higher quantity of orbital fat was removed during the procedure [58] (Table 1).

### 3.8. Radiographic Changes

Sharma et al. retrospectively reviewed CTs and MRIs of patients before and after treatment with GLP-1 RA, measuring midface volume craniocaudally from the inferior orbital rim to the inferior area of the hard palate and dividing the area into nine superficial and deep compartments, with the infraorbital and cheek fat being included in the superficial and the soborbicularis oculi fat (SOOF) being included in the deep compartments. They found that patients lost 11% of the superficial midface volume, which was statistically significant, while losing 7% of deep facial volume, which was not significant. According to the results, a patient can expect to lose, on average, 7% of the volume of the midface for every 10 kg of weight that they are losing. This finding is different from radiographic changes related to the ageing process, which showed that there is significant volume loss in both the deep and superficial compartments [59,60].

Imaging modalities, like MRI or ultrasound, have been suggested to be potentially beneficial in assessing the fat distribution, changes in deeper tissues, and tissue integrity, giving valuable insights to practitioners to tailor the treatment for each patient [61].

### 3.9. GLP1R Agonists’ Effects on Ocular Surface

In a study in a murine model of diabetes mellitus type 1, expression of GLP1-RA receptors was identified in the lacrimal gland. Furthermore, it was shown that hyperglycaemia caused fibrosis and atrophy of the lacrimal gland, which resulted in reduced tear secretion. Interestingly, liraglutide was applied topically and reduced the fibrosis and inflammation of the lacrimal gland, improving the tear production [62].

The treatment of type 2 diabetes mellitus (T2DM) patients with GLP-1 RA was associated with improvement in tear film stability and production. In the study of Ottonelli et al., DMT2 patients who were treated with GLP-1 RA were two groups of type 2 diabetic patients, with one of them being treated with GLP-1 RA showed significantly longer tear break-up time (TBUT) and higher Schirmer I test measurements compared to patients who were treated with other antidiabetic medications, though there was no significant change in the Ocular Surface Disease Index scores between the groups. One of the limitations of the study was that the antidiabetic medications of Sodium–Glucose Cotransporter-2 SGLT2 inhibitors that were used by the patients in the control group might have reduced the tear production. The authors, therefore, suggested that treatment with GLP1-RA can potentially reduce the risk of dry eye disease [63]. A similar protective effect in dry eye disease with treatment of diabetic patients with GLP1-RA was found in the study of Pan et al., though they also concluded that treatment with SGLT-2 inhibitors was also protective in dry eye disease [64].

In a retrospective population-based study, Fan et al. found that treatment of T2DM patients with GLP-1 RA was associated with a significantly lower incidence of superficial keratitis and dry eye disease, which was more pronounced in patients younger than 60 years old. They recommended preferential treatment of T2DM patients younger than 60 years and at risk of dry eye disease with GLP-1 RA due to their protective effect [65].

However, in a retrospective study that used a multi-institutional database in Taiwan, it was found that SGLT2 inhibitors were associated with a lower incidence of dry eye disease in DMT2 patients compared to treatment with GLP1-RA, which contradicts the results of the study of Ottonelli et al. [66] (Table 2).

**Table 1:** GLP1 RA effects on Ocular Adnexa.

Study (Author, Year)	Country	Study Design	Sample Size	Population Characteristics	Methods	Key Outcomes	**Notes/Limitation**
Humphrey et al., 2023 [37]	USA	Narrative review	4 included studies	N/A	Facial effects and perioperative considerations of GLP-1 RA	Describes mechanism of rapid weight loss leading to “Ozempic face”, includes clinical implications, surgical and aesthetic management and anaesthesia considerations	Expert opinion level evidence, no primary data
Montecinos et al., 2024 [38]	USA	Comprehensive review	46 included studies	N/A	Facial changes caused by semaglutide	Similar changes to a naturally aging face but in people much younger	Comprehensive review, no primary data
Carboni et al., 2024 [39]	USA	Narrative review	N/A	N/A	Review of literature on GLP1-RA, facial fat loss, elastin changes and clinical implications for dermatologists	Facial sagging likely due to rapid weight loss in diabetic and non-diabetic patients and reduction of elastin due to aging. Need for accurate history to clarify GLP1-RA usage and inform patients on effects.	Narrative review, no primary data
Sarlos et al., 2025 [40]	Brazil	Case report/case series	2 patients	-Patient 1: 32-year-old woman who had rapid weight loss due to semaglutide -Patient 2: 32 year-old-man who had rapid weight loss due to semaglutide	Both patients were injected with PLLA-SCA in the upper and mid-face, then had filler HA injections 30 days later and then a second session of PLLA-SCA 15 days later.	Both had improvement in skin quality, Ogge’s curve, submalar shadow, contour of malar area and increased palpebral aperture. No significant adverse effects. Suggestion of early biostimulation with PLLA-SCA for patients being on semaglutide.	Limited generalizability as only two cases. No standardized measurements of palpebral aperture.
Tay, 2023 [41]	UK	Editorial/Commentary	N/A	Describes general population using semaglutide for weight loss and particular concerns in middle-aged adults with pre-existing collagen decline	Literature review and author commentary	Effects of facial fat loss, hollowing, sagging, deterioration of rhytids. Affected areas: tear troughs, temples, cheeks and lower face. Treatment with dermal fillers, autologous fat transfer, laser and energy devices, cessation of semaglutide though metabolic health concerns.	Opinion/letter, no patient cases or testing of intervention.
Widgerow, 2024 [42]	USA	Narrative review + Expert opinion (Level 5 evidence)	N/A	N/A	Comprehensive review on dWAT, adipocytes, fillers, GLP1-RA effects on fat. Summary of biological mechanisms.	GLP1-RA may inhibit adipocyte differentiation causing reduced dWAT volume, “Ozempic face” features, increased fibrosis.	No new experimental data or new clinical trials.
Ridha et al., 2024 [43]	Canada-USA	Narrative review	N/A	Patients using GLP1-RA for diabetes or weight loss	GLP1-RA effects on adipose tissue and stem cells, muscle mass changes, clinical signs of “Ozempic face”, evaluation of regenerative treatments	GLP1-RA reduce dWAT and collagen production and impair ADSC proliferation, therefore facial aging not solely due to weight loss. Also muscle loss. Management with ADSC-targeting modalities like PLLA, fat grafts, HA fillers.	No patient data, relies on mechanistic evidence.
Rahman et al., 2025 [44]	International	Content, sentiment, social network analysis	15 studies reviewed, 1 survey report, Social media posts analyzed	General public, patients, influencers, practitioners discussing GLP1-RA treatments online	40% increase in filler consultations related to GLP1-RA aesthetic effects. Regional differences. Ethical concern as practitioners who prescribe GLP1-RA are also offering filler treatments	No primary clinical measurements, cross-sectional	
Chen et al., 2025 [45]	USA	Case series	36 participants	18 patients taking GLP1-RA and 18 matched control patients	Measurement of several distances of facial landmarks on digital photos using the software	No significant change in upper face distances of facial landmarks with GLP1-RA, only significant change in orolabial measurements of female participants.	Patients on GLP1-RA were recruited from an Oculofacial clinic, therefore they had mainly oculoplastic complaints with half of them having upper dermatochalasis and half of them ptosis
Burke et al., 2025 [47]	USA	Narrative review	N/A	Diabetic and non-diabetic patients using GLP1-RA for diabetes or obesity respectively	Review of mechanistic studies, therapeutic uses, dermatologic adverse events	Facial fat loss from rapid weight loss associated with GLP1-RA. Improve wound healing	No primary experimental data
Sharma et al., 2025 [59]	USA	Retrospective cohort study	24 patients	Patients who were treated with GLP1-RA and had head and neck CT/MRI both before and after the treatment	Measurement of changes in total, deep and superficial volume	Patients lost 11% of the superficial midface volume which was significant while losing 7% of deep facial volume which was not significant. Patients can expect a loss of 7% of midface volume for every 10 kg of weight that they are losing.	Quantitative prospective analysis that proves the “Ozempic face” effect. Small sample and not all patients received the same imaging modality (CT/MRI)
Haykal et al., 2025 [61]	France	Clinical commentary, review and clinical observations	N/A	Patients who have rapid weight loss due to GLP1-RA for obesity or metabolic treatment	Review of literature and clinical observations assessing aesthetic effects and possible management options	Rapid weight loss associated with GLP1-RA causes loss of facial volume and skin laxity. Potential usage of ultrasound or MRI imaging to tailor treatments. Treatment with dermal fillers, fat grafting, biostimulating fillers, energy based devices like RF (in lower face)	Not primary research

**Table 2:** GLP1 RA effects on Ocular Surface.

Study (Author, Year)	Country	Study Design	Sample Size	Population Characteristics	Methods	Key Outcomes	Notes/Limitation
Ottonelli et al., 2025 [63]	Italy	Retrospective, single center, case-control study	35 patients (21 on GLP1-RA and 14 on non-GLP1-RA	Adults with T2DM, median age 73 years with a median T2DM duration of 4.05 years	Assessment of tear production and stability with Schirmer test and TBUT, OSDI questionnaire. Comparison between GLP1-RA versus non-GLP1-RA users	Improved tear production showed with higher Schirmer 1 test values and longer TBUT in GLP1-RA users	Retrospective study, small sample. Potential confounding effect of SGLT2 inhibitors.
Su et al., 2025 [64]	Taiwan	Retrospective population based cohort study using a multi-institutional Research Database	152,520 patients	Adult patients with T2DM excluding patients with prior ocular disease or no anti-diabetic treatment	Data from Chang Gung Research Database from 2005 to 2020. Assessed demographics, HBA1c, ocular procedures.	GLP1-RA were found to have a protective effect for dry eye disease compared to metformin	Retrospective design
Fan et al., 2023 [65]	Taiwan	Retrospective population-based cohort study	6021 patients (2007 GLP1-RA users and 4.014 non-users)	T2DM patients excluding patients with pre-existing dry eye disease or keratitis	Data from the Taiwan National Health Insurance Research Database from 2014 to 2020	Lower incidence of superficial keratitis and dry eye disease in patients younger than 60 years who were treated with GLP1-RA	Retrospective design, potential confounding effect of concurrent anti-diabetic medications
Su et al., 2022 [66]	Taiwan	Retrospective population-based cohort study	1077 patients using GLP1-RA and 10,038 patients using SGLT2 inhibitors	Adults with T2DM newly receiving GLP1-RA or SGLT2 inhibitors excluding patients with pre-existing dry eye disease or ocular comorbidities	Data from Chang Gung Research Database analyzed from 1 March 2022 to 31 May 2022	Lower incidence of dry eye disease of SGLT2 inhibitors compared to GLP1-RA	Much larger sample size of patients receiving SGLT2 inhibitors compared to GLP1-RA

### 3.10. GLP1R Agonists Effects on Skin

The GLP-1 peptide is considered to cause the preadipocytes to regress to myofibroblasts and fibroblasts, affecting the dermal white adipose tissue (dWAT), which is located at the base of the hair follicles and in the dermis layer, and is associated with several physiologic processes. This effect can lead to atrophy of fat, wrinkling, and sagging [42]. GLP1-RA can cause loss of elastin, collagen, and nutrients in the skin, which is more noticeable in older patients who have a reduction in these elements already. Furthermore, they can lead to dryness, as they affect the skin barrier.

### 3.11. Management—Informed Consent

In the review of Rehman et al., only 38% of the included studies reported a consistent practice of updating patients on the aesthetic side effects of GLP-1 during the consultations [44]. A thorough consultation should take place with medical professionals before prescribing weight loss medication to clearly discuss the risks and benefits, so that patients have realistic expectations regarding their aesthetic changes [37,38].

One option could be to discontinue the medication, which could lead to weight regain, which has been termed “Ozempic rebound,” and can be a treatment option, though often less favourable [14,38].

### 3.12. Management with Fillers

A significant increase in consultations for fillers has been noted in Europe and North America for aesthetic concerns due to GLP-1 treatments [44]. It has been suggested that restoring the volume of the midface and malar area with fillers can be the first step to achieve a positive effect on the neighbouring tear trough area, which can then be injected with hyalouronic acid (HA) filler if needed [67]. Placement of HA filler in deep midfacial layers can cause stretching of the fibroblasts and activation of ADMSC, which can change the extracellular matrix and improve appearance [67].

A 40% increase in filler consultations related to GLP-1 cosmetic issues has been noted, which is largely attributed to influencers who recommend them. Ethical considerations have been raised due to dual-role providers, who prescribe both weight loss medication and aesthetic interventions, due to a potential conflict of interest [44].

The mechanism of volume loss improvement with fillers includes two mechanisms, which are the direct physical filling and the stimulation of collagen type 1 and elastin synthesis. They can last from 1 month to 5 years, but the elastin and collagen induction can offer a longer-lasting improvement [38].

In case the individuals regain weight after GLP-1 medication cessation, potential filler dissolution may be needed as their natural adipose tissue regenerates around the face, which can be problematic with the more permanent fillers [39].

Periorbital hollowness, though not specifically related to weight loss, has been successfully treated with injections of HA filler in the orbital rim, septal area, zygomatic area, or eyebrow and cheek fat pads, reshaping the 3-dimensional appearance of the periorbital complex [68].

Treatment of a patient with enophthalmos due to anatomically enlarged posterior–inferior orbital cavities with HA filler injections into the extraconal and inctraconal posterior orbit has been described, which improved his globe position and upper lid hollowness; although, as previously mentioned, there are no studies that identified enophthalmic changes due to GLP1-RA usage [69].

### 3.13. Management with Poly-L-Lactic Acid (PLLA-SCA)

PLLA-SCA is a biodegradable, biosynthetic polymer that can remodel the extracellular matrix, modulate the responses of macrophages, and stimulate the production of type I collagen. It is considered a regenerative restoration treatment and usually requires 3–4 sessions of injections, and its onset of results is gradual and can last up to 2–3 years [61].

A multi-centre study assessing the treatment of patients who had weight loss from GLP-1 RA with injections of poly-l-lactic acid (PLLA-SCA) and hyaluronic acid fillers found a significant improvement in contours of the face and fullness of cheeks. The researchers concluded that PLLA-SCA can provide a long-lasting improved outcome due to elastin and collagen regeneration, while HA fillers help with instant volume optimisation [70].

PLLA-SCA was injected into the subcutaneous layer of the temporal, zygomatic, malar, and tear trough areas, among other areas, of two patients who lost significant weight over 5–6 months while being on semaglutide. They had a second injection session of PLLA-SCA 45 days after the first one, and an injection of HA fillers 30 days after the first session. It was noted that there was an improvement in skin quality, contour of the malar area, Ogee’s curve, and submalar shadow, and increased palpebral aperture. The positive outcomes are due to the combination of PLLA-SCA with HA fillers. The authors suggest early initiation of biostimulation with PLLA-SCA, even before significant weight loss, and then maintenance injections [40].

### 3.14. Management with Surgical Procedures

Regarding the timing of a surgical intervention, it is important to take into consideration the loss of nutrients, which can include B12, iron, protein, and fat-soluble vitamins; thus, the surgery should be postponed until nutrition is optimised and the patient has achieved and maintained their weight loss goal [37].

The delayed stomach-emptying should always be considered in planning a surgical procedure. A brow lift can achieve the repositioning of the adipose tissue. Surgical procedures can be augmented with fat grafting.

With their radiographic study, which demonstrated more significant volume loss in the superficial compared to the deep compartments, Sharma et al. suggested that treatments should be focused more on the superficial tissues and can include rhytidectomies and treatment of the superficial fat compartments with fat, and also Botox treatments [59].

Two patients showed delayed wound healing and fat necrosis after undergoing breast surgery and while being on GLP1-RA. One of them kept using GLP1-RA, while the other stopped the medication one week before her surgery. These complications were potentially explained by the reduced mechanical support and vascularity due to the fat oxidation caused by the GLP1-RA. The authors suggest increased vigilance of the effects of GLP1-RA in elective surgeries [71].

However, it has been reported that GLP1-RA may have beneficial properties in the healing of diabetic patients and diabetic wounds [47].

Considering the fat loss due to GLP1-RA, surgeries, including blepharoplasties that restore and recontour tissue, might be preferable. Specifically, blepharoplasties with fat repositioning or fat grafting, which can be combined with skin tightening if there is skin laxity [72,73].

### 3.15. Management with Fat Grafting

Autologous fat grafting can be performed on its own or to augment a surgical procedure. It involves harvesting fat and then injecting it into hollow areas or rhytids [37,38]. Its efficacy in treating aesthetic outcomes due to GLP-1 therapies remains uncertain, as, in theory, the grafted fat becomes metabolically active and can then undergo the same catabolic process as native fat, which can influence its longevity [44].

## 4. Discussion

GLP-1 receptor agonists, which are used for diabetes and the management of obesity, have gained attention for aesthetic applications due to their significant weight loss effects. Rapid facial and periocular fat loss associated with these medications can lead to functional and cosmetic ocular changes, necessitating awareness for multidisciplinary care and patient counselling [37].

The periocular region is particularly vulnerable to visible changes due to its thin dermis, minimal subcutaneous fat, and reliance on deep and superficial fat pads for contour. The preferential loss of superficial midface fat seen on imaging aligns with clinical reports of more pronounced tear troughs, hollowing, and increased rhytids [59]. It is not clear if GLP-1RA directly influences the survival of adipocytes, but plausible mechanisms include altered preadipocyte differentiation and reduced adipose-derived stem cell viability. These changes resemble typical ageing-related atrophy, but appear in a younger age [37,40,42].

Unlike superficial facial fat, orbital fat is structurally specialised and metabolically distinct. Current evidence does not support consistent orbital fat depletion with GLP-1RA therapy. However, due to cases of enophthalmos after severe weight loss, it might be useful to have future radiologic studies focused on orbital tissues [48,49,55]. It is important to clarify these potential changes, as globe position influences eyelid support, tear distribution, and surgical planning.

Interestingly, GLP-1RA may have beneficial ocular surface effects despite their aesthetic drawbacks. Preclinical data demonstrating reduced lacrimal gland fibrosis and enhanced tear production suggest an anti-inflammatory or anti-fibrotic mechanism [62]. Clinical studies support potential improvement in tear film metrics, though results are confounded by comorbidities and concurrent medications [63,64,65,66]. Understanding these interactions is essential in preoperative evaluation for blepharoplasty and ptosis repair, as ocular surface health and tear film stability can be affected by these procedures [74].

As GLP-1RA use expands beyond endocrinology, plastic and oculoplastic surgeons increasingly encounter patients seeking correction of GLP-1-associated facial changes. Management should prioritise accurate assessment of superficial versus deep fat loss, stabilisation of weight, optimisation of micronutrient status before surgery, and a multimodal approach combining fillers, biostimulatory injections, and surgical contouring [40,44,45,67,70].

The unique pattern of superficial atrophy suggests that interventions targeting superficial fat compartments may be particularly effective. However, long-term outcomes of autologous fat grafting remain uncertain due to the potential susceptibility of grafted adipocytes to ongoing metabolic effects. Clear communication with patients is essential, and there is a need for improved interspecialty communication. Patients pursuing weight loss for health or cosmetic reasons may be unprepared for the unintended consequences of rapid fat depletion, creating dissatisfaction and additional financial burden [37,38,44].

### 4.1. Limitations of Current Evidence

The existing literature is limited by heterogeneity, small sample sizes, reliance on retrospective studies, and a predominance of anecdotal or observational reports. Radiologic data remain scarce, and few studies quantify changes in periocular or orbital parameters with standardised measurements. Similarly, ocular surface findings require clarification through prospective, controlled studies that isolate the effects of GLP-1RA from potential confounding effects of other antidiabetic therapies.

### 4.2. Conclusions—Future Directions

While there is a general sense in the surgical and aesthetics communities that treatment with GLP1-RA causes significant facial changes, which can include hollowness and fat atrophy in the periocular, temporal, tear trough, and cheek area, and loss of fat in the subcutaneous layer of the temples and cheeks, there are only a few studies that accurately measure these changes.

Most of the studies that assess the aesthetic facial changes related to GLP1-RA are quite recent and from the USA, while the ones assessing ocular surface changes related to GLP1-RA usage are from Taiwan.

Longitudinal studies that address the long-term periocular, orbital, and ocular surface changes due to GLP-1 treatments are needed. The changes can be assessed with high-definition photographs, accurate 3-dimensional measurements, or CT and MRI scans using a similar methodology to studies that assess ageing with these modalities. Furthermore, studies that assess autologous fat-grafting longevity and resistance to lipolysis and case series of patients treated with GLP1-RA who had eyelid rejuvenation surgeries would be beneficial. Likewise, prospective studies that investigate any alterations of the tear film and dry eye incidence in patients on GLP1-RA for weight loss would be beneficial, as it is a significant preoperative factor in decisions related to blepharoplasty and ptosis repair.

A potential future area of research could be to assess the effects of the emerging Nutrient Stimulating Hormones (NuSHs) and Myostatin–activin pathway inhibitors (MAPi), which are currently being investigated for their potential to preserve muscle during pharmacologic weight loss on facial musculature.

## Data Availability

No new data were created or analyzed in this study.

## Abbreviations

The following abbreviations are used in this manuscript:

GLP-1RA	Glucagon-like peptide-1 receptor agonists
GLP-1R	Glucagon-like peptide-1 receptor
GIP	Glucose-dependent insulinotropic polypeptide
ADA	American Diabetes Association
DR	Diabetic retinopathy
NAION	Non-arteritic anterior ischemic optic neuropathy
AMD	Age-related macular degeneration
POAG	Primary open-angle glaucoma
OHT	Ocular hypertension
dWAT	Dermal white adipose tissue
ADSCs	Adipose-derived stem cells
SOOF	Soborbicularis oculi fat
T2DM	Type 2 diabetes mellitus
TBUT	Tear break-up time
SGLT2	Sodium–Glucose Cotransporter-2
PLLA-SCA	Poly-L-Lactic Acid
HA	Hyaluronic acid
